# Potentially burdensome care at the end-of-life for people who had a death from cancer: A retrospective population-level cohort study

**DOI:** 10.23889/ijpds.v9i5.2481

**Published:** 2024-09-18

**Authors:** Victoria Udalova

**Affiliations:** 1 U.S. Census Bureau

Direct data collection via surveys of patients and health care providers continues to experience declining response rates and increased costs. Alternative data sources such as medical claims or electronic health records hold significant promise to complement direct data collection efforts but these records often suffer from a lack of important sociodemographic, economic, and other critical social determinants of health characteristics. On the heath care provider side, existing records often lack information needed to fully describe complex organizational relationships between providers and their places of work, employment, and service delivery. Through strategic partnerships with external data owners, the Enhancing Health Data (EHealth) program at the U.S. Census Bureau links multiple data sources of restricted administrative records covering patients, providers, and health care businesses and leverages data assets and record linkage capabilities available exclusively at the Census Bureau. This presentation will describe existing EHealth projects and share the vision to build a data infrastructure that empowers researchers, policy makers, and the public to uncover new insights on patients, providers, and population health.

